# Longing for normalcy in couple relationships: How chronic illness and care dependency change the relationship of long-married couples

**DOI:** 10.3389/fpubh.2023.1117786

**Published:** 2023-03-17

**Authors:** Katharina Niedling, Kerstin Hämel

**Affiliations:** Department of Health Services Research and Nursing Science, School of Public Health, Bielefeld University, Bielefeld, Germany

**Keywords:** chronic disease, marital dyad, qualitative research, long-term care, Germany, spouses, home nursing, caregivers

## Abstract

**Introduction:**

Coping with chronic illness and care dependency in a marital dyad challenges many older couples. In our qualitative research study, we are interested in how long-married spouses in Germany experience their couple relationship while dealing with long-term care and adapting everyday life to the care situation.

**Methods:**

We conducted problem-centered interviews with 17 spouses according to the interpretive-reconstructive documentary method.

**Results:**

We derived four thematic areas: (1) partner(ship) disappears behind the disease; (2) partners struggle with changing tasks and roles; (3) caring partners mourn the loss of intimacy; and (4) partners strive to rebalance the partnership.

**Discussion:**

When chronic illness and care dependency enter couples' lives, the self-image as husband or wife is affected. Primary health care professionals should be sensitive to the specific constellation of care in couple relationships and recognize the significance of this dyadic relationship as living in a satisfying couple relationship is essential for the health and wellbeing of both partners.

## 1. Introduction

For A population of 4.1 million people is care dependent in Germany (1). Approximately 80% of them are aged 65 and over, and the risk of care dependency grows exponentially with increasing age (1). Overall, approximately four-fifths of people who are in need of long-term care live in their own household; only ~36% of them use home care services (1). Close relatives shoulder the majority of care responsibilities. In the case of older people who live in a partnership, (spousal) partners are the first to assume the task of care (2). (Spousal) partners account for approximately one-third of the informal primary caregivers in Germany (2). This constellation of care is currently also favored by the 1930 to 1950 birth cohort showing a “historically unique marriage rate with a comparatively low risk of divorce” (3) in Germany. However, the care provided by partners in long-term marriages has received little attention in long-term care policies and nursing research in Germany (4).

Progressive functional impairments and the need for care gradually change couples' daily lives and partnerships (5); it is crucial for them to adapt and maintain daily routines, such as shared mealtimes (4, 6). Persson et al. (7) elaborated in their study that continued attachment in their dyadic relationship is perceived by both partners as a lived experience of dignity in palliative care. However, older couples who constitute a care relationship are less likely to involve other relatives in daily care than caregiving daughters and sons (2). Couples are also more skeptical about involving home care services, as they fear disruptions to their well-established daily routines and private spheres (8, 9).

However, caring partners are usually themselves of advanced age and in poor health (10, 11). Caregiving responsibility often becomes a heavy burden in a relationship characterized by both partners' high vulnerability (12–14). There is also some evidence that the health limitations of one partner can negatively influence the other partner's satisfaction with the marriage relationship (15). Other studies point out that increased responsibility and care for an ill or impaired partner can lead to more closeness and intimacy, which in turn have a positive effect on marital satisfaction and thus subjective wellbeing and health (11). “Good marriages” are associated with a lower risk of mortality and cardiovascular disease for the partners (16). However, it must be kept in mind that the decision to care for one's partner is made independently of the relationship's quality (12). So the couples need help and home visits by nurses could provide this. But for this, it is important to understand couple relationships.

In couple relationships, there is a sense of belonging to each other. Couples form a common identity and a knowledge of their “we.” Couple rituals, as symbolic actions, also make up bonding dynamics (17). Partners maintain their social or institutional order as a couple, for example, through relationship symbols, such as joint anniversaries and gifts. According to Lenz (17), a collaborative relationship culture is constitutive for couples, in which a joint reality is created and maintained through communication and negotiation between the partners. Couples negotiate joint norms over time. They create a culture of intimacy that also serves as a boundary to the outside world (18). This boundary is consistent with the fact that only a particular perception of the partner and the couple relationship is “allowed.” A “high degree of commitment (exclusivity)” (17) as well as “an increased degree of attention” (17) within the relationship often exists, especially for long-term partnerships. The continuation of the couple relationship creates stability and security for both partners affected by the chronic illness of one of the partners (11). According to Willi (18), a couple relationship's success is also based on the partners' experience of equal roles within the partnership.

Changes such as those that occur in care situations interfere with these ways of functioning (19), which yield a constant imbalance between giving and receiving (20–22). Conflicts arise when couples can no longer maintain former agreements, roles and power relations. Luitgard Franke (6) speaks of ambiguity as a “reversible figure” (“Kippfigur”), especially in the context of a partner's dementia. This figure describes how, in some situations, the marital relationship becomes dominant, while in other situations, the caring relationship's characteristics become dominant (6). Such an interplay of roles and power structures leads to conflict for many couples. In the study “What keeps marriages together?” by Klaus Schneewind et al. (23), conflict competence is identified as a necessary “keystone” of marital relationships. Schneewind et al. examine functional and dysfunctional conflict resolution strategies. Constructive problem solving is described as making compromises, which contrasts with dysfunctional strategies such as verbal aggressiveness and saying nothing. Sociologist Georg Simmel [see (24)] sees conflicts as necessary for negotiating social orders. He ascribes a function of reordering to conflicts. According to Simmel (24), people who are very close to one another can sense “how insignificant [the conflict] is in relation to the unifying forces” (24). Indifference in conflict, on the other hand, points to a lack of depth of feeling. Couple sociologist Lenz (17) also describes conflicts as having a stabilizing function.

For health care providers, like nurses, it is important to have knowledge about the life world of the person in need of care, as the life world led care approach of Dahlberg and Todres (25, 26) emphasizes. In this context, it is important to focus not only on the health and wellbeing of the patient but also on the health and wellbeing of the caring partner, because the care situation is a critical life event and a specific health risk for both partners.

However, the interplay of relationship and care dynamics in long-term couples in a domestic care context as well as the resulting conflicts or the joint handling of conflicts remain insufficiently researched.

Against the background of the challenges that older couples face in simultaneously constituting a couple and a care relationship with each other, our study aims to more closely understand how partners proceed to “integrate” (or not) both sides of their relationship.

What does it mean for the couple relationship when the care dependency of one partner and a care relationship manifests in long-term marriage?

How do long-married partners experience their couple relationship while dealing with chronic illness and adapting their everyday lives to a care relationship?

## 2. Materials and methods

Problem-centered interviews, following Witzel (27), with biographically oriented narrative stimuli were conducted with heterosexual long-married couples in Germany in which one partner cared for the other; data analysis was conducted using the interpretative-reconstructive documentary method (28, 29). The standards for reporting qualitative research (SRQR) (30) were followed in this study.

### 2.1. Interview guide

The interview guide themes were developed by the first author in a preliminary study based on three group discussions (31), each with eight to nine nursing professionals working as so-called care trainers. These nurses guided and accompanied family caregivers in home care in the project “Family care under the conditions of the G-DRG” (32). In this respect, the nurses had special expert knowledge (contextual knowledge) about the target group of couples in care relationships. In the group discussions (31), the nurses were asked to share their views on older couples' living situations and living environments in care relationships. The identified themes were then fed into the guideline questions, which were further developed based on three pretests. The resulting interview guideline included the following topics: relationship history, roles within the couple relationship, couple rituals, care networks, needs and need satisfaction within the relationship, changes in physicality/intimacy, care motivation in and coping strategies with the care situation, daily routines, sleeping situation and changes in the home.

The problem-centered interviews (27) began with an open invitation (33) to move toward a narrative interview style (“Tell me how you became a couple and how things have developed between you up to the present?”). All interviewed couples first traced a picture of their relationship history and common stations in life. Only then did they begin to describe the care situation and share more details on various topics through focusing (follow-up) questions. For the interviewer (first author), it was important to listen actively and thus support the participants' narratives. The follow-up questions also served to initiate narratives, specifically, to describe everyday situations and the couple's relationship and to maintain the flow of the conversation. The participants determined the course of the conversation and set topics independently in most cases. Biographical data that could not be concluded from the interview were collected in person or by telephone after the interview.

### 2.2. Sampling and field access

We mainly recruited couples or caring partners who participated in care courses in the project mentioned above, “Family care under the conditions of the G-DRG” (32). The first author invited the couples to participate in the study while attending the courses and via the nurses who conducted these courses; the participants were informed about the aims, subject matter and study procedure both verbally and in writing via informational flyers.

The criteria for selecting the couples were as follows: the partners were 65 years or older, had established a long-term marriage, lived together in one household, and one of the two partners was care dependent with the other partner described as the main caregiver. In this study, living in a long-term marriage was not defined by years of marriage but rather by the partners sharing a long common couple history as well as emotional intimacy. This includes a structure of roles, functions and tasks within the partnership that has been consolidated over many years (34, 35). Similarly, it was not of interest whether the care dependent partner was eligible for Long-Term Care Insurance benefits; it was only relevant whether the couples self-reported a long-term care situation.

We conducted ten interviews (= cases) with 17 persons from December 2018 to June 2020. As far as possible, interviews with couples were attempted (36). For ethical reasons, the study participants were free to decide whether they would participate in the interview as a couple or whether each partner would complete an individual interview. As a result, three interviews took place with only the caregiving partner (all women); seven interviews were conducted with both partners present. Eight interviews were conducted in the couples' homes, and one was conducted in a separate area of a hospital café that is intended for counseling sessions with relatives. In one case, a telephone interview was held due to the ongoing COVID-19 pandemic.

The sample is characterized by a wide range of heterogeneous and contrasting (couple) situations and diagnoses, as experienced by health professionals in their practice. In two cases, the carer was male; in eight cases, the carer was female. The duration of the marital relationships ranged from 30 to 63 years, and the duration of the care relationships ranged from 1 to 30 years. Table 1 presents a sample overview. The interviewees—both the cared for and the caring partners—reported a range of functional limitations and chronic diseases with which they were affected. In four cases, the care partners had been diagnosed with dementia at different stages of the disease.

**Table 1:** Sample overview (*n* = 10 couples).

	**Duration of care < 5 years**	**Duration of care over 5 years**
Duration of relationships 30–40 years	2 couples	1 couple
Duration of relationships >40 years	2 couples	5 couples

The interview partners ranged in age from 65 to 95 years. The age difference of the partners was a maximum of 3 years in seven out of ten cases, 7 years in two cases and 20 years in one case. In all cases, the male partner was older. With one exception, all couples had at least one child together. Four couples had an intermediate education level in common, and four couples had a higher education level. In two other cases, the husbands had a university degree, and the wives had an intermediate education. All of the couples lived in urban areas in different parts of Germany. All couples lived together, nine in their own houses (of different sizes), and one had moved from their owned house and lived in a rented flat due to the husband's care needs.

### 2.3. Ethical considerations

Before and after the interviews, the interviewer provided verbal and written information about the study and the processing of personal data. Written informed consent was obtained from all study participants, who were informed that the interview could cause strong emotions and that they could take a break or end the interview at any time. If an interviewee lived with cognitive limitations, the information was given in easily understandable language (37).

Persons living with dementia were explicitly included to conduct research with them rather than about them. Especially in the initial phase of the disease, dementia patients are capable of providing information about subjective everyday experiences, life situations and needs (38).

During the interview, the interviewer carefully considered and decided whether comprehension and focussing questions could be asked or whether doing so would intrude on the couples' privacy. For example, questions about the couples' sexuality were not explicitly addressed; rather, the topic was approached under the heading of “intimacy/physicality.” In almost all of the conversations, the interviewees, both the caregivers and the cared-for partners, shed tears, revealing the couples' vulnerability. At these particularly sensitive points in the conversation, the interviewer paused until one of the interviewees began to speak again of his or her own accord.

### 2.4. Data analysis

The interviews lasted ~1.5–2.5 h. They were audio-recorded, fully transcribed and pseudonymised. Data analysis was performed using MAXQDA software. The documentary method used is an interpretative-reconstructive content analysis method through which collective orientations and habitus are resolved (28, 29); this method was particularly suitable for analyzing the couples' interaction processes. In line with the ideal-typical step-by-step procedure, a case-by-case analysis was performed (39). In the first step, each interview's content was described, i.e., the transcript was segmented into content sections. In the second step, segments were identified that indicated (potential) conflicts within the couple relationship due to the care situation. The selected segments include passages where the interview partners explicitly described conflicts as well as those where conflicts were implicitly communicated because the imbalance of the relationship became particularly clear at these parts. The latter were particularly present in narrative passages where a joint, seamlessly connected partner narrative came to a standstill and/or interviewees ceased “cooperative reporting” as “we” or speaking to one another; in other situations, conflict was indicated by the interviewees expressing themselves uncooperatively or sometimes competitively and contradictorily to one another. In this way, how the interviewees expressed their conflicts in the interview was investigated. The respective segments were analyzed in depth to investigate potential underlying conflict. For the interviews conducted with one partner, the interview passages in which conflicts and differences of opinion were made (more or less) explicit by the interviewee were analyzed in greater depth.

In the third step (formulating interpretation), statements included in these segments were described in the analyser's words to gain an initial analytical distance from what had been said. In the fourth step (reflective interpretation), the interaction style between the spouses (and the interviewer) was described for the selected passages. Based on this, it was finally possible to present a course of discourse and a case or discourse description for each case. The final type formation of the documentary method, as recommended by Bohnsack et al. (28), was not carried out for this presentation of results. Instead, selected sequences from one interview were compared with sequences from other interviews in relation to the overall course of the interview to identify similarities and differences.

The first author performed the segmentation and analysis of the data material according to the above steps and presented the data material for discussion several times in research workshops during the analysis. The team of authors condensed the thematic fields by in-depth interpretation of the data.

## 3. Results

Overall, we derived four thematic areas that describe how the partners experience their couple relationship while dealing with chronic illness and adapting their everyday life to a caring relationship: 1) partner(ship) disappears behind the disease; 2) partners struggle with changing tasks and roles; 3) caring partners mourn the loss of intimacy; and 4) partners strive to rebalance the partnership.

### 3.1. Partner(ship) disappears behind the disease

The interviewees described the course of their couple relationships as increasingly “disrupted” by the progress of chronic illness and functional limitations. Daily life as a couple is shaped by diseases and limitations. Most of the interviewees perceived illness and subsequent care needs primarily as a phenomenon of natural age(ing) that crept into their relationship:

“*We really grew into this situation very, very slowly, without really noticing it. And at some point, it was clear when there was a problem to solve. Yes, the partner is no longer there, he can't say anything about it, and then that was it” (Mrs. Kühl, carer)*.

The possibility of “growing into” the new situation together makes it easier for the couple to deal with it. According to most of the interviewed caring spouses, they have (thus far) been able to cope with the care situation and the requirements they face more or less “adequately.” Simultaneously, however, they find it almost intolerable when the partner “disappears” behind his or her illness, for example (but not exclusively), in the case of dementia. Their well-established relationship as a couple seems to fade away:

“*Because it doesn't happen abruptly, you can endure it well. You just learn. But the fact that you then have a “child” is a bit more difficult, but it works. (sighs) It works. But the couple relationship is–totally gone” (Mrs. Stuck, carer)*.

The caregiving spouses feel increasingly alone coping with everyday problems and decision making. They now also have to make decisions for and about the partner. They perceive an enormous loss of partnership, while the cared-for partners also complain about the loss of their autonomy. Restrictions due to impairments dominate couples' lives in many ways, and the way they deal with conflicts in the partnership also changes. When both were still in good health, the partners seemed to be “compatible” with one another; for example, they actively “fought out” their conflicts:

“*When at some point it became too much, then a clear word was spoken and, well, then the steam was out, and then it was good again, until next week” (Mrs. Kühl, carer)*.

As time passes, arguing costs the couples too much strength and energy; in some cases, it is simply no longer possible for the ill partner to actively engage in conflicts:

“*I just scold him, and he doesn't have much to counter it with. Nope. No, he can't anymore. So, he doesn't have the words either. And you notice how incredibly exhausting it is for him to get a message across to me. And when the words are missing and all that, in such an emotional situation, […] then nothing works. And so that he doesn't fall silent completely, we'd better leave it altogether, right?” (Mrs. Teusen, carer)*.

An open conflict becomes too stressful and emotionally overwhelming, especially for partners requiring care. The couples know one another well and know which issues cause arguments, so now they must decide whether it is necessary to deal with the conflict. Consequently, many of the carers switched to avoiding conflicts or not addressing them openly to protect the weakened partner. The cared-for partners describe themselves as rather restrained and try to avoid potential conflicts as much as possible. They also see this as necessary because they do not want to create an additional burden for their partner:

“*I myself tend to take it easy in everyday life and wouldn't cause any trouble if I wasn't pushed to the limit” (Mrs. Falke, cared-for)*.

### 3.2. Partners struggle with changing tasks and roles

The interviewed couples had an established division of tasks and roles in daily life in their long-term relationships, as one caregiver observed: “*The division was clear. I just do it; it always went well*” *(Mr. Schwarz, carer)*. In most cases, they follow a traditional role model typical for their generation in which the wife primarily takes care of the household and family members and the husband focuses his responsibilities outside the house and on financial management, as one husband noted, “*But cooking and such things were not my concern at all” (Mr. Falke, carer)*. The partner, in his or her role as caregiver, increasingly takes on the responsibility that used to be reserved almost exclusively for the partner in need of care.

Mr. Falke complains about finding himself in an unusual, even inappropriate situation as a man while caring for his wife, an interpretation that his wife opposes:

*Mr. Falke (carer): Yes, well, if you know people, right, statistically speaking, the man dies 5 years before the woman*.

*Mrs. Falke (cared-for): And with us it's the other way round, do you think? I have ALWAYS nursed you in the years BEFORE, you*.

Due to the impaired partner's withdrawal from tasks and responsibilities, power relations begin to change. Caring partners, especially women, are given more power within the relationship and, as a result, show themselves to be more dominant. Some express feelings of stability and security in taking over this responsibility; it adds meaning to his or her life. Others, however, find it burdensome that they must set the tone for the couple's daily life, noting: “*It's terrible that I now have such a say over you” (Mrs. Liszt, carer)*. Losing their own area of responsibility is a great hurdle for the cared-for partners, as one husband observed: “*And now, I have nothing more to say” (Mr. Schwarz, cared-for)*. Some perceive this giving up of responsibility as an intervention by the partner that goes too far, and the partners show how discordant their view of this is, as shown in the following conversation:

*Mrs. Böge (carer): Yes, right, and in addition, I can't do that anymore, because my husband can't. He helps as much as he can, but it just doesn't work anymore*.

*Mr. Böge (cared-for): He's not allowed to do it anymore*.

*Mrs. Böge (carer): Stop it with your “I'm not allowed anymore”!*

Even persons who feel relieved to hand over responsibility to his or her partner remain skeptical about whether the partner can do the job as well as they can. As a result, carers often feel that their work is not valued. Both partners no longer feel that they are in an equal relationship with one another; rather, it is painful for both of them to acknowledge their new roles.

### 3.3. Caring partners mourn the loss of intimacy

Only a few of the interviewed couples talk about intimacy or sexual life, with only caregiving spouses talking about this in more detail. For Mrs. Schmöning (carer), it is painful to no longer be able to satisfy her own needs and desires for intimacy, as she explained:

“*That's what hurts me. Where I think you can hold on to that even into old age if you stay healthy, right?*”

“*I don't allow closeness anymore, and neither do you. We don't talk about that either!” (Mrs. Schmöning, carer)*.

Mrs. Stuck openly expresses her dissatisfaction about a lack of “*kindness”* toward her partner. Addressing her husband, she complains that frailties in other couples have not necessarily led to less intimacy and tenderness:

“*I'm sometimes jealous of people who go for walks holding hands, older people like that. Maybe they can't do more than just go for a walk. But they do it and walk holding hands” (Mrs. Stuck, carer)*.

Similarly, other carers emphasize that they no longer perceive themselves as wives or husbands in their relationship. Rather, it is often only about “functioning” in daily life, and they subordinate their relationship to the illnesses and the associated impairments, observing:

“*You are only in between the nurse and the housewife and nothing more. Anyone else could have done that just as well. That's how I felt sometimes. Someone else can do it, someone else can do it, right?” (Mrs. Kühl, carer)*.

The changed relationship appears to be similar to one between siblings or between a mother and child, as one carer explained: “*Because I'm practically caring for you now, like one actually cares for a child or something” (Mrs. Liszt, carer)*. In their stories about everyday caregiving, she refers back to childcare terms *(“...I always say, the “Pamper,” “Stopper socks””—Mrs. Liszt*).

### 3.4. Partners strive to rebalance the partnership

Despite the challenges of having to adjust to a changed relationship, most of the interviewed persons seem to have found a sustainable way of being together as a couple because “*you work together” (Mrs. Kühl, carer)*. The partners accept a kind of dependency on one another, which they perceive as unavoidable. However, the different ways of dealing with the care situation cause both partners to experience feelings of being left alone with their problems so that sometimes they cannot see a way out. Therefore, they cannot help but tease one another, despite the tense situation: “*Sometimes you tease each other more often because you don't have an outlet” (Mrs. Hampel, carer)*. Simultaneously, the partners see one another and their roles in a new way, which at times results in viewing the situation as an opportunity to find new balance in the relationship:

“*That was actually noticeable when my husband no longer wanted to do what he used to be able to do, but when he could no longer do everything, we were somehow more attuned to each other” (Mrs. Teusen, carer)*.

In some cases, however, the partners reach different conclusions about their relationship:

*Mrs. Schmöning (carer): It's true, a lot of things have crumbled. At least for me. And my husband doesn't talk to me, so I can't say how it is with him. He says it's all good*.

*Mr. Schmöning (cared-for): It is*.

*Mrs. Schmöning (carer): Nope. Not for me*.

*Mr. Schmöning (cared-for): But I think so*.

## 4. Discussion

Our study's aim was to understand how older, long-married couples perceive their couple relationship when they constitute a care relationship. We were interested in analyzing the experiences by the exploration of “dyadic” or couple narratives' (40). Most of the interviewed persons in our study accepted the invitation to talk together as a couple about their everyday lives and changing couple relationships, from which four themes emerged.

*(Partner)ship disappears behind the disease:* The interviewees describe their couple relationship and daily life as sometimes interrupted while adapting to and coping with illness and functional limitations. If the illness becomes increasingly dominant, they sense a fading away of their partnership, to some extent as a creeping process but often combined with a (late) moment of realizing its definite point; both cared-for and caring partners interviewed in this study express feelings of being left alone despite living together. The caring partners described, as did the study participants in the study by Sorber et al. (41), the burden of bearing everything alone and of being solely responsible for the functioning of daily life together. In both studies, the couples emphasize that the joint life and the individual life are dominated by the illness of one partner. In her study of married couples in which one person is living with dementia, Franke (6) describes that the couples initially avoided openly talking with each other about the disease and its consequences for the couple relationship because it seemed too threatening to them. When the symptoms of the disease eventually become apparent, the couple's interaction with each other is disturbed and changes drastically insofar as less communication takes place at the couple level. Our findings show a similar pattern for couples dealing with cognitive impairment.

*Partners struggle with changing tasks and roles:* In accordance with other studies (5, 15, 23), our findings show how partners struggle when they have to give up their “taken for granted” tasks and roles in everyday life due to illness and care dependency. As Abendschein et al. (5) suggest, partners long for normalcy, but it costs them a great deal of strength to handle the changes due to the care situation. Accordingly, the persons interviewed in our study perceive changing tasks and roles as a particular burden, as it irritates their self-concept as a husband [also see (42)] or wife. Lenz (17), following a rather traditionalist view of couple relationships, sees a close link between division of labor in the household and the corresponding distribution of power within two-partner relationships. In accordance with this, our study indicates a change of power relationship in the marriage in that the caring partner becomes more powerful if the sick person has to permanently withdraw from daily duties. For example, caring women of the generation who are mostly financially dependent on their husbands take over finance management and thus gain power within the relationship. However, the new tasks and roles in managing everyday life can be perceived by the women as either an enrichment [also see (6)] and empowerment or as an enormous burden (43).

*Caring partners mourn the loss of intimacy:* Only the caregiving partners in our sample talk about the loss of intimacy in the couple relationship. Since the need for care brings harshness and an intrusion into the intimacy of the partner, an important pillar of the couple relationship is lost. Although some studies [see (6, 11)] suggest that caring for an ill or impaired partner may lead to a special intimacy or closeness, our study does not show this. Consistent with the study by Sorber et al. (41), some of our interviewees report role changes toward the role of a “nurse.” Others describe their relationship as a sibling or mother-child relationship. They describe that they no longer feel perceived as spouses, which, as Sorber et al. (41) study showed, goes hand in hand with a decrease in sexual activity.

*Partners strive to rebalance the partnership:* The partners in our sample sometimes vent about small disputes. The pressure of the mismatch of give and take, as Boszormenyi-Nagy and Spark (20) also explain, can be seen as a trigger for that. Bödecker (22) describes giving and receiving help as a core conflict in this context. This also has the effect that a functioning couple relationship, in which balancing care for the other and the self is essential [see (19)], is difficult to establish for the couples in our sample. The couples balance autonomy and commitment, determining and being determined. For both partners in these couple relationships, there is a strong dependence on each other due to the care situation (9, 13). According to Bödecker (22), the partner in need of care must “at least not fundamentally deny” his or her need for help and accept his or her own dependence to a certain degree so that the relationship can find a new balance. As our study shows, finding a new balance between focussing on the illness and the relationship can be understood as a developmental task for both partners. However, working together as a dyad on the couple relationship is made more difficult by the health limitations of one partner.

### 4.1. Theoretical conceptual considerations

The relationship of couples in which one partner is in need of care cannot be continued as it was before the onset of the disease, and relationship dynamics inevitably change, which can be seen very clearly in the example of managing conflicts. The lost intimacy the caregiving partners of the sample describe corresponds to the no longer working boundaries to the outside world described by Willi (18). However, we noted how the caring partners strive to maintain this boundaries by refusing help and trying to stick to routines. Relatedly, the specific view of the partner being cared for described by Willi (18) is also lost. This view only focussed on certain characteristics of the partner and transfigured others. This also affects the equal roles described by Willi.

In line with other studies [ex. (6, 44)], our analysis shows that the interviewed couples struggle to reconcile the couple and the care relationship. Unlike Franke (6), however, we cannot observe a tipping figure according to which, once the couple relationship and once the care relationship become relevant. Both relationships always intertwine and take place simultaneously. We observed that the couples in the sample worked on integrating the care relationship into the couple relationship and on changing the couple relationship. Thus, previous rituals, which Lenz (17) describes as constitutive of the relationship, are not abandoned but adapted. A shared reality, as accomplished according to Lenz (17) via communication and negotiation between the partners, and continuity are disrupted. Life as a couple as it was before the onset of the chronic illness no longer exists for most couples.

### 4.2. Strengths and limitations

With this study, we were able to reconstruct the relationship dynamics of couples in dyadic narratives. This was possible because not only what was described by the couples was analyzed, but also what was not carried out, as well as unfinished narratives or subtly carried out conflicts in the interviews. The dyadic narrative in interviews offers a suitable instrument for future research with couples in care situations, in order to be able to tap into the dynamics that unfold in relationships between two people. However, our study also has limitations. A large proportion of the interviewees had already accepted help in the form of trainings, home visits and consultations on the care situation within the framework of the project “Family care under the conditions of the G-DRG” (32) and thus was used—to a certain extend to talk about their care situation. Furthermore, the sample represents a certain “positive selection” of interview participants for whom it can be assumed an already better balancing of care and couple relationships than in the case of couples without this support. A broader sampling strategy could enable a more diverse picture in future research, including couples who no longer manage to rebalance. Another weakness of our study is that most of the couples belonged to the upper educational class, which suggests a higher capacity for reflection and better linguistic skills than for groups with a low level of education. Future research should also focus on couples from lower socioeconomic statuses; moreover, other methods, such as observations, could offer further insights into the study's objectives.

### 4.3. Implications for health professionals working with older couples in care relationships

This study highlights the unique situation of long-married couples who are slowly entering into a caring relationship—a quite common care constellation in aging populations but rather neglected by public health and long-term care policies. The uniqness is characterized by the simultaneity of coping with care and the couple relationship—two aspects that ultimately influence the health and wellbeing of both partners. Health professionals working with older couples should be aware of this potentially for both partners overstraining constellation. They should be able to comprehensively address not mere practical care needs but strengthen resources by supporting the couples to take care of their relationship despite a demanding care situation. Psychosocial support, as well as promoting mental health of both partners are fundamental here.

Primary health care professionals, especially nurses as frontline care workers involved in home care situations, are particularly “close” to the couples (45, 46). They should work together with the couple in a lifeworld-oriented manner to strengthen the mental health of both partners and accompany them in their specific relationship dynamics. With the increasing complexity of relationship entanglements in the course of the care situation, it can be challenging to deal with these care dyads. Especially in challenging cases, where the health of both partners may be at risk, it is recommended to work in an interprofessional team with professionals with special- (mental health, gerontological etc.) expertise (47), as is the case in a mental health team, for example. Close cooperation between nurses and social workers (48) can be just as helpful as consultation with or referral to psychotherapists—for both partners. In the past, it has become apparent that health professionals focus too much on the illness and the patient, while the coping measures of partners and families are primarily focused on the disturbed life (49). In terms of a lifeworld orientation, aspects such as role development should be adequately included (50). This also includes helping to resolve conflicts rather than glossing over them (51). When working with couples, professionals should go beyond patient-centered care and aim for relationship-centered care (9, 52). The burdened caregiver's existing resources and wellbeing should also be supported (53). The availability and needs orientation of health services and the accessibility of these services need to be improved in this context (54), as couples are among the most isolated care constellations.

## Data availability statement

The datasets presented in this article are not readily available due to the potentially revealing nature of complete interview transcripts. Requests for access to the datasets should be directed to katharina.niedling@uni-bielefeld.de.

## Ethics statement

Implementation of this study was approved by the Ethics Committee of Bielefeld University (No. 2021–181). The assessment was carried out in accordance with the ethics guidelines of the Deutsche Gesellschaft für Psychologie (German Psychological Society) and the Berufsverband deutscher Psychologinnen und Psychologen (Professional Association of German Psychologists). All study participants gave their written informed consent beforehand. The patients/participants provided their written informed consent to participate in this study. Written informed consent was obtained from the individual(s) for the publication of any potentially identifiable images or data included in this article.

## Author contributions

KN conceptualized and designed the study, collected, analyzed and interpreted the data and drafted the manuscript. KH conceptualized and designed the study, interpreted the data and drafted the manuscript. All authors read and approved the final manuscript.
